# Radiological Correlates of Raised Intracranial Pressure in Children: A Review

**DOI:** 10.3389/fped.2018.00032

**Published:** 2018-02-23

**Authors:** Saeed Kayhanian, Adam M. H. Young, Rory J. Piper, Joseph Donnelly, Daniel Scoffings, Matthew R. Garnett, Helen M. Fernandes, Peter Smielewski, Marek Czosnyka, Peter J. Hutchinson, Shruti Agrawal

**Affiliations:** ^1^School of Clinical Medicine, Addenbrooke’s Hospital, University of Cambridge, Cambridge, United Kingdom; ^2^Division of Academic Neurosurgery, Department of Clinical Neurosciences, Addenbrooke’s Hospital, University of Cambridge, Cambridge, United Kingdom; ^3^Department of Neuroradiology, Addenbrooke’s Hospital, University of Cambridge, Cambridge, United Kingdom; ^4^Department of Paediatric Intensive Care, Addenbrooke’s Hospital, University of Cambridge, Cambridge, United Kingdom

**Keywords:** brain, injury, ONSD, transcranial Doppler ultrasound, intracranial pressure, basal cisterns

## Abstract

Radiological assessment of the head is a routine part of the management of traumatic brain injury. This assessment can help to determine the requirement for invasive intracranial pressure (ICP) monitoring. The radiological correlates of elevated ICP have been widely studied in adults but far fewer specific pediatric studies have been conducted. There is, however, growing evidence that there are important differences in the radiological presentations of elevated ICP between children and adults; a reflection of the anatomical and physiological differences, as well as a difference in the pathophysiology of brain injury in children. Here in, we review the radiological parameters that correspond with increased ICP in children that have been described in the literature. We then describe the future directions of this work and our recommendations in order to develop non-invasive and radiological markers of raised ICP in children.

## Introduction

Traumatic brain injury (TBI) in children remains a UK and worldwide public health concern. Early management of TBI aims to prevent secondary brain injury and invasive monitoring of intracranial pressure (ICP) plays an important role of the management of pediatric neurocritical patients (1).

The gold-standard for ICP measurement requires an invasive intraparenchymal monitor. Although generally regarded as safe, this procedure carries a small risk of hemorrhage, infection, and seizures (2–5). In patients with coagulopathies, invasive monitoring may be contra-indicated. Furthermore, in the global context of neurosurgery, a requirement for the expertise to insert such a device can result in delays in the implementation of guided medical therapy: some areas of the world are served by 1 neurosurgeon per 9 million patients (compared with the 1 per 80,000 in developed countries) (6). These issues have been brought to the fore by the results of a recent randomized-control trial in adults, which questioned the ostensible positive effect that invasive monitoring has on outcomes, stimulating debate as to whether invasive monitoring is over-utilized in current practice (7).

As such, an accurate and reproducible methodology for assessing raised ICP would be highly beneficial and allow for stratification of which patients would benefit from invasive monitoring. While radiological features are already recognized and used in clinical practice to alert to raised ICP (for example: midline shift, ventricular effacement, sulcal effacement, cistern effacement, and herniation), these features have not been extensively validated in a pediatric cohort.

Here in, we review the radiological parameters that correspond with increased ICP in children that have been described in the literature. We then describe the future directions of this work and our recommendations in order to develop non-invasive and radiological markers of raised ICP in children.

## Studying ICP in Children

The anatomical, physiological, and pathophysiological differences between children and adults mean that specific pediatric studies are essential in validating proposed radiological correlates of raised ICP.

**Figure 1:**
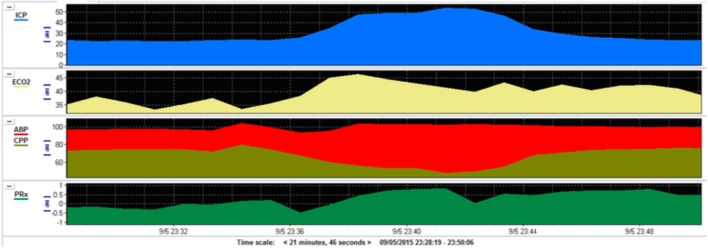
Multimodal monitoring during plateau waves in intracranial pressure (ICP) after traumatic brain injury (TBI). This is a pediatric patient who sustained severe TBI with moderate diffuse axonal injury (Marshall grade 3) on initial computed tomography scan. During the monitoring on the pediatric intensive care unit the patient developed rapid and short-lived increases in ICP (blue) in a pattern known as intracranial plateau waves. This corresponds with a reduction in cerebral perfusion pressure (CPP, gold) and a deranged cerebrovascular autoregulation (PRx, green). PRx—pressure reactivity index—is a measure of the capacity of the cerebral vasculature to alter its resistance in response to changes in CPP. A negative PRx indicates intact pressure reactivity whereas a positive PRx indicates impaired pressure reactivity.

In adults, intracranial hypertension (IH) is defined as an ICP that is persistently raised above 20 mm Hg (8). In children, normal values are age dependent. While there is continued debate on age directed strategies, the consensus is that brief increases in ICP that return to normal in <5 min may be insignificant; however, sustained increases of ≥20 mm Hg for ≥5 min should likely warrant treatment (9) (Figure 1).

Compared with adults, children may be more likely to develop diffuse brain swelling after TBI (10). This has been postulated to be because of immature or impaired autoregulation of cerebral perfusion pressure, an enhanced inflammatory response, and increased blood–brain barrier permeability in the developing brain (11, 12).

Children have a lower mean arterial blood pressure. This means that if a child does develop intracranial hypertension, they may be more likely to have a critically decreased cerebral blood flow (CBF) and thus sustain a secondary ischemic injury (13). There is extensive debate on whether management of acute brain injury should be targeted by ICP thresholds, by CPP thresholds or both. Only a small number of pediatric studies have demonstrated CPP-directed intervention. In two studies, the lower limit of the scale that was used was 40 mm Hg (14, 15), and in two other studies, it was 45 mm Hg (16, 17).

Furthermore, the biomechanical differences between adult and children’s skull in relation to the brain offer different levels of accommodation. In infants, the presence of fontanelles allows for buffering of raised ICP (18).

## Imaging Modalities

The imaging modalities that have been tested against ICP are computed tomography (CT), magnetic resonance imaging (MRI), and ultrasonography (US). CT is routinely performed in children with suspected IH and so parameters in this modality are of importance in terms of immediate clinical utility. However, the lack of ionizing radiation with MRI and US make these modalities attractive alternatives, given that children are more radiosensitive and have longer life-expectancies compared with adults (19). However, MRI is rendered difficult with an uncooperative and distressed child due to long acquisition times and may be contraindicated in major trauma with potential metal foreign bodies. Furthermore, while US is available at the bedside, avoiding the hazards of patient transfer, and is radiation-free, it is not routinely used for this purpose in the context of TBI and there is limited, albeit growing, experience in this field.

## Radiological Parameters

A multitude of radiological parameters have been examined in children within small cohorts.

### Basal Cisterns

**Figure 2:**
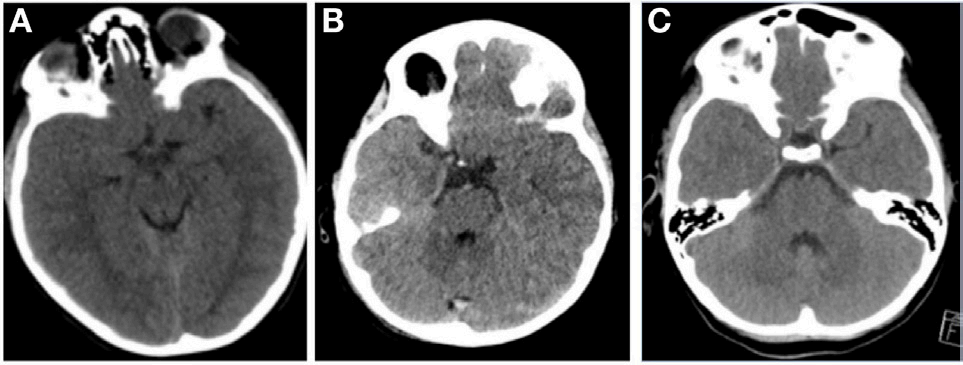
Representative images of pediatric patients with raised intracranial pressure (ICP). **(A)** A patient with acute subdural hematoma (ASDH), opening ICP 32 mm Hg. **(B)** A patient with diffuse axonal injury, opening ICP 25 mm Hg. **(C)** A patient with ASDH, opening pressure 28 mm Hg. All these pediatric patients demonstrate open basal cisterns, despite pathologically raised ICP.

The appearance of compressed or obliterated basal cisterns on CT images and its correlation to elevated ICP has been well studied in adult cohorts (20). Kouvarellis et al. have found that this correlation also holds true in children, with 75% of their cohort who had obliterated cisterns demonstrating at least one episode of elevated ICP on invasive monitoring (21). However, they also found that the presence of open cisterns does not necessarily correspond to normal ICP, with open cisterns having a positive predictive value of only 59% in detecting an ICP below 20 mm Hg. This is an important finding: an observation of patent cisterns in the presence of raised ICP can have significant implications for management of such patients. As such, a defined threshold of basal cistern compression in relation to raised ICP would be helpful to interpret this data. Moreover, the pathophysiology of this finding is interesting in itself. Given the close relationship with the pediatric brain with the inner table of the cranium, it is surprising that pathological ICP can be accommodated this efficiently, without displaying cardinal radiological signs of hypertension (Figure 2). Advances in understanding the mechanism of how the pediatric brain combats these fluctuations in pressure following injury may have multiple implications for CSF disorders.

### Optic Nerve Sheath Diameter

**Figure 3:**
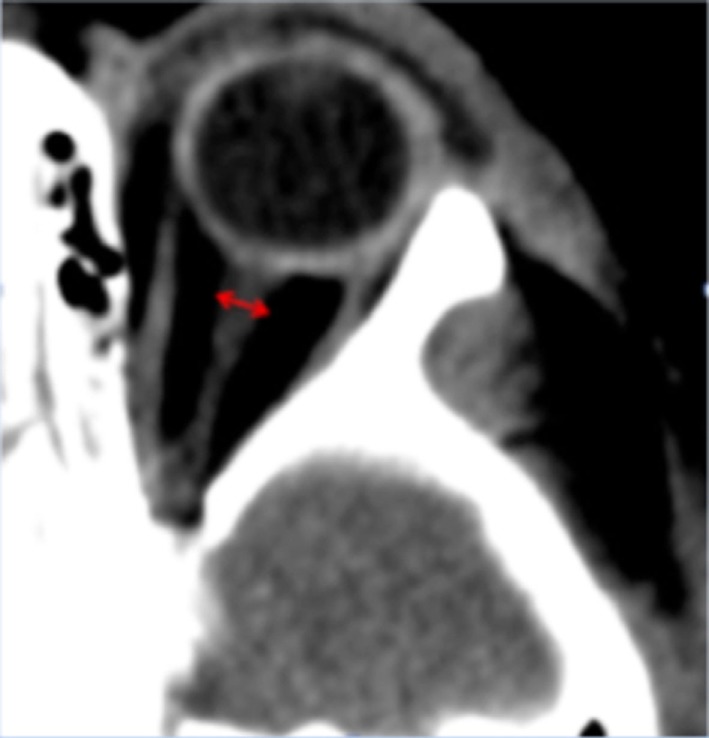
Representative computed tomography image of a pediatric patient showing measurement of the ONSD. The ONSD is typically measured 3 mm behind the insertion of the optic nerve into the globe, perpendicular to the long axis of the optic nerve.

The optic nerve sheath communicates with the subarachnoid space of the meninges and its diameter has been shown to widen in the context of elevated ICP (Figure 3) (22, 23). This phenomenon has been demonstrated to occur within minutes of acute changes in ICP and thus the ONSD poses an attractive target for non-invasive ICP monitoring (24).

Ultrasonography measurement of widened ONSD has been investigated as an indicator of elevated ICP in adults (25–27). In children, this correlation has also been demonstrated in a number of studies, with the cohort of 174 children in Padayachy et al. (largest to date) exhibiting a sensitivity of 80% in detecting ICP ≥ 20 mm Hg (28). However, the reliable use of US may require technical expertise that is not widely available.

Only one study has explored the viability of ONSD in relation to CT values in pediatric cohorts (29). This study achieved a much-improved specificity (91%) than similar studies in adult cohorts [42%, in one recent adult study (30)] to detect elevated ICP. It has been postulated that this difference may be as a result of children’s brain parenchyma being intimate with the cranial vault, without the deep sulci that develop in late adolescence—meaning that changes in ONSD are subject to less inter-patient variation (28). This highlights the importance of deliberate pediatric studies in this field, with a variation in anatomy manifesting as a clear difference in neuroimaging parameters. The effectiveness of ONSD in a modality that is routinely acquired in current practice is of significant interest, although this being a single-center, small sized study means validation in larger cohorts is necessary.

A comparison of MRI to CT in measuring ONSD suggest that measurements should be in close agreement across both modalities and, indeed, Hirfanoglu et al. have recently demonstrated this potential correlation for children (31).

Thus, ONSD would seem a reliable parameter for evaluating ICP in children, being available in modalities of CT that is routinely used to diagnose TBI, and in US that reduces radiation exposure and minimizes time transferring patients from safety of the ICU. It is worth noting however, that the relationship between ONSD and raised ICP is dependent on establishing and validating threshold values above which ICP is considered elevated. The studies in children to date have been retrospective analyses that have used cutoff values that maximize the specificity and sensitivity of their measurements. It will be more difficult to adopt these cutoff values prospectively, given the interindividual variability in children of different ages in particular, but also of different genders and ethnicities (32, 33).

### Intracranial Elastance

An emerging technique for measuring ICP using MRI is by using the concept of intracranial elastance. Elastance is defined as the ratio of change in pressure to change in volume, and an elastivity index has been determined for the brain over a range of ICPs (34). MRI analysis of CSF velocities and arterial, venous and CSF flow volumes are used to calculate the small fluctuation in intracranial volume and pressure change during the cardiac cycle, which is then related to ICP using the known relationship between ICP and elastance (35).

There have been some studies to assess this model in adults but to date the only application in pediatric cohorts comes from Muehlmann et al. who found a positive correlation (Spearman ρ = 0.64, *p* < 0.01) between shunt opening pressure and MR-ICP in 15 children with hydrocephalus (36). Based on these observations there is reason to suspect that there is a potential role for intracranial elastance measurements in pediatric TBI patients. The technique is likely to be too cumbersome and time-consuming to provide rapid diagnosis and aid with decision-making criteria on therapy. However, there is a wide scope to use the modality to gain a greater understanding of the pathophysiology of intracranial hypertension following TBI and gain an insight into potential therapy.

### Cerebral Blood Flow

Measurements of CBF can be studied using two modalities, transcranial Doppler ultrasound (TCD) magnetic resonance angiography (MRA). To date, only TCD has been used in the context of pediatric TBI.

Measurements of CBF by TCD rely on the observed physiological phenomenon that elevated ICP leads to a greater reduction in diastolic flow velocity than systolic flow velocity (37). This relationship is exploited by calculating the Gosling pulsatility index (PI), defined as the difference between systolic and diastolic flow velocities, divided by the mean flow velocity (37). The results of PI correlation to ICP in adults have shown limited utility, with numerous studies concluding that the relationship may only be reliable at extreme values of ICP (38, 39). However, in children, this relationship may be of more value. Notably, the findings of O’Brien et al. suggest an extremely good correlation in the very early stages postinjury, with ICP ≥ 20 mm Hg being predicted with 100% sensitivity and 82% specificity (40). This relationship does not seem to hold true more than 24 h postinjury, but this may still render TCD a valuable tool in screening which patients require invasive monitoring in monitoring for secondary brain injury (41).

Although MRA has not been demonstrated in pediatric TBI, the technique has been used in pediatric hydrocephalus patients. Measurement CBF at the level of the internal carotid artery and basilar artery were performed with the conclusion of only a moderate correlation (*r* = −0.55) with raised ICP (42). However, significant reduction in CBF would be expected to require severe elevation in ICP. The study was performed in infants (age range = 1 day to 7 months old) who were young enough to have open fontanelles. This makes it more likely, therefore, that these patients would have been able to tolerate significant rises in ICP before exhibiting any clinical signs. Indeed, Bateman, failed to reproduce this correlation in a cohort of older children (mean age = 8 ± 5) (43). As such, given the complexity of the analysis and the time delay in image acquisition and analysis, MRA is unlikely to provide parameters that would be clinically useful in pediatric TBI.

## Future Directions

A growing body of evidence is demonstrating some potentially beneficial modalities for using radiological parameters to guide therapy in pediatric TBI. Early work has already identified some thresholds to improve both sensitivity and specificity of such radiological markers.

There are existing radiological classification systems in adults, such as Marshall or Rotterdam scores, that have shown value in correlating radiological evidence to predict outcome (44, 45). While some of these have been validated in children there is scope to refine this to better suit the pathophysiology of pediatric TBI (46). A study combing the outlined measurements above would evaluate whether they serve to direct care more efficiently.

A validation of the radiological parameters of raised ICP on CT imaging would be of the most immediate clinical value, given this modality’s widespread use in current practice. Currently, the parameters from adult studies are assumed as valid—an assumption that has already been questioned, as discussed above. Moreover, given the possible age-related differences (e.g., as a result of open fontanelles or changes in skull compliance), it would be prudent for future studies to stratify their pediatric cohorts by age, rather than group children as one demographic as some previous studies have done. Given the number of potential variables involved a large, prospective study specific to children would allow for validation of the most suitable radiological markers.

## Author Contributions

All authors listed, have made a substantial, direct and intellectual contribution to the work, and approved it for publication.

## Conflict of Interest Statement

The authors declare that the research was conducted in the absence of any commercial or financial relationships that could be construed as a potential conflict of interest.
